# Analytical and Experimental Study on Fluid–Solid Coupling of Variable-Caliber Nozzles for Concrete 3D Printing

**DOI:** 10.3390/ma19040695

**Published:** 2026-02-11

**Authors:** Lianzhi Zhang, Xiao Li, Lin Lin, Changzai Ren, Yibo Wang, Kun Yang, Sen Xue, Linlin Fei

**Affiliations:** 1 School of Mechanical Engineering and Automation, Liaoning University of Technology, Jinzhou 121000, China; 13864147189@163.com (L.Z.); lntu163@163.com (Y.W.); yangkunwh@163.com (K.Y.); wyxx2157@163.com (S.X.); 2 Department of Engineering Practice Education, Liaoning University of Technology, Jinzhou 121001, China; 3 School of Intelligent Manufacturing, Liaoning Polytechnic University, Jinzhou 121000, China; 18841671827@163.com; 4 School of Energy and Power Engineering, Qilu University of Technology, Jinan 250316, China; rcz@qlu.edu.cn; 5 State Grid Jinzhou Power Supply Company, Jinzhou 121000, China; blue080@163.com

**Keywords:** concrete 3D printing, variable-size nozzle, fluid–solid coupling, rotating blade structure, surface quality

## Abstract

Concrete 3D printing technology is emerging as a new way to transform the construction industry in the future. However, the existing concrete 3D printing technology still has different degrees of defects in the print molding process. The existing concrete 3D nozzles need to undergo a long motion trajectory when printing complex curved components, which leads to lower geometric accuracy of curved structures, as well as poorer overall molding quality of the printed components. The aim of this study is to design a reducer nozzle to effectively shorten the printing stroke and thus improve the printing accuracy. A reducing nozzle is proposed with multi-gear internal meshing and a rotating blade structure nozzle with an adjustable outlet caliber. The mechanical strength of the rotating blade of the nozzle and the distribution characteristics of the flow field inside the nozzle are verified through fluid–solid coupling analysis. Experimental comparison shows that compared with the existing concrete 3D printing nozzle, the variable-caliber nozzle significantly improves the surface quality of the specimen, which strongly promotes the practical application and development of concrete 3D printing technology in the engineering field.

## 1. Introduction

With the rapid development of the construction industry in the direction of intelligence and automation, concrete 3D printing technology has become an important research direction for the industrialization of construction due to its advantages of moldless construction, high design freedom, and significantly reduced construction costs [1,2]. Although the current concrete 3D printing technology has developed rapidly and made certain achievements, most of the current printing nozzle sizes are fixed. When using a fixed-geometry nozzle for narrow to wide mutation area printing, it is often necessary to carry out multiple cycles of repeated printing [3], or reside in a large surface for a long time, in order to obtain the matching print molding components [4], and the concrete material has its own rheology, which can produce slumping or lateral expansion due to self-weight or too short a time between prints without curing [5]. The traditional methods of printing are difficult to apply directly. This directly reduces the efficiency of printing. Secondly, the limitations of the size, shape, and mechanical capability of existing reducer nozzles may lead to manufacturing errors [3].

One of the key challenges facing concrete 3D printing technology is the surface quality defects of molded specimens and the “overfilling” and “underfilling” problems caused by fixed cross-section size nozzles [6]. As an important index for evaluating the 3D printing process, improving surface quality has become a key direction of current research. In order to address this problem, scholars at home and abroad have put forward a variety of solutions: Tu, H.; Wei team [7] innovatively used a lateral scraper mechanism to mechanically trim the extruded material, which effectively improves the surface quality, but the additional device restricts the degree of freedom of the printing; Roussel [8], from the perspective of rheology, proposed optimization of the material rheological properties to reduce the deviation of the extruded concrete morphology and the size of the nozzle; Kwon et al. [9] systematically investigated the influence law of nozzle outlet geometry on surface quality; Motamedi et al. [10] used a six-axis robotic tilt-printing technique to improve the surface quality of curved structures but was limited by a fixed nozzle size, which was only able to optimize the surfaces on both sides of the specimen. A study by l. Hashmi, A.W [11] demonstrated that precisely controlling the size of the printing nozzle is essential to improve the final surface quality. In the absence of effective control, the concrete material is prone to deformation under the influence of gravity and rheological properties [12], thus affecting the surface quality.

The main defect of the existing fixed-size nozzle is that it is difficult to match the morphology of the printed specimen to the shape of the design model, and the curved structure presents a discrete approximation of the morphology [13], which not only limits the molding quality but also makes it difficult to ensure the overall construction quality [14]. To solve this problem, this paper proposes a 3D printing variable-caliber nozzle, the advantages of which are reflected in the compact structure, low inertia, and circular outlet. This is accomplished by adjusting the nozzle caliber size to ensure that the concrete print specimen meets the design size requirements. This optimizes the print path planning, significantly shortening the print stroke. Compared with the traditional fixed-caliber nozzle, it can improve the molding quality of the print specimen. The problems of “overfilling” and “underfilling” are effectively avoided. The experimental results show that this technology can effectively improve the surface quality and dimensional accuracy of complex structures, providing a new solution for the engineering application of concrete 3D printing technology.

## 2. Reducing Nozzle Structure

**Figure 1:**
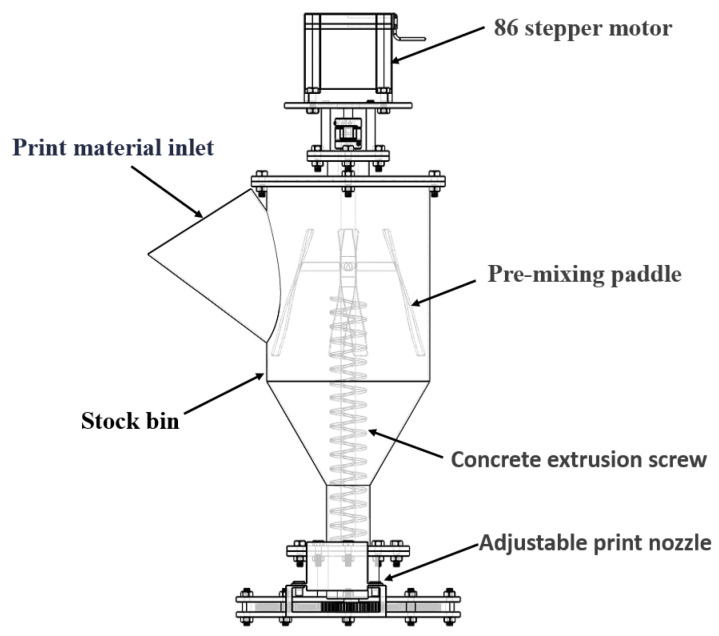
Reducing nozzle structural unit.

Given the diversity and limitations of existing nozzle designs, this solution employs a central variable-caliber structure. This variable-caliber nozzle effectively shortens the printing stroke, thereby enhancing printing precision. Experimental comparisons demonstrate that this structure markedly improves specimen surface quality compared to existing concrete 3D printing nozzles. Its architecture comprises multiple sets of identical rotating blades paired with pinions, arranged in alternating close formation. The gear meshing mechanism enables adjustable nozzle operation. The design concept employs a detachable variable-speed solution, with nozzle components connected via bolts. Each part can be replaced independently, with multiple components combining to form a complete nozzle assembly [3]. Adjustable speed refers to the capability to control mortar extrusion rate by regulating the screw rotation speed. To address the quality defects in forming with existing fixed-caliber nozzles, this paper proposes an adjustable-caliber concrete 3D printing nozzle. Its overall structure is shown in Figure 1.

### Caliber Adjustment Module

In a reducer nozzle, parameters such as blade type, number, and width have a direct impact on performance in terms of adjustment range, accuracy, and flexibility; therefore, rotary blade selection is a key issue to be addressed in the design [15].

Taking into account that the rotating blade thickness has a greater impact on the overall thickness, according to the size of the through-hole, we selected blade thickness of 2 mm and a 304 stainless-steel plate. The boundary of the extruded caliber is a curved polygon formed by the circular arc of the blade, and the number of its sides is the number of blades used. The increase in the number of blades allows the boundary of the caliber through-hole to be closer to a circle, which theoretically improves the equivalence with a standard circular caliber plate [16]. In view of the viscosity of concrete and the sensitivity to the roundness of the caliber boundary, and considering the comprehensive manufacturing, installation and use aspects, 24 blades were selected. The specific parameters are shown in Table 1.

**Figure 2:**
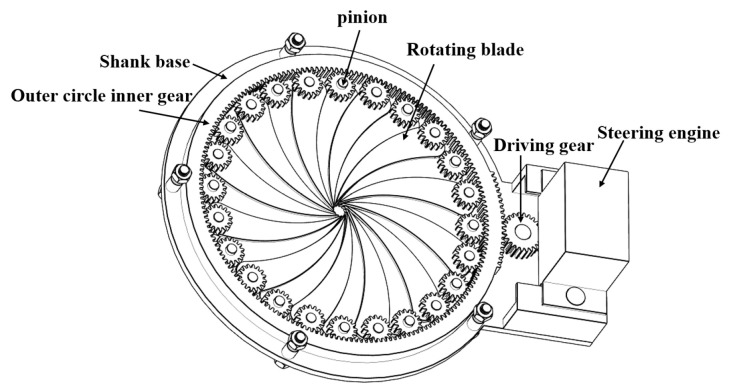
Diagram of the internal assembly of a reducer nozzle.

In this paper, the adjustable nozzle part of the structure shown in Figure 2. The actual work of the nozzle relies on the helm top gear and the active gear meshing for rotation, thus driving the other end of the meshing of the inner gear for rotation. The inner gear and pinion gears mesh with each other, thus driving the pinion and rotary vane fixed in the positioning column assembly so that it will be involved in the printing process. By changing the rotary vane rotational angle, one can change the shape to circular. The size of the nozzle caliber is changed by changing the angle of rotation of the rotary vane during the printing process. Moreover, the aggregate particle size in the concrete used for the experiment typically ranges between 1.5 and 4 mm, with larger particles being more prone to clogging within the nozzle channel. The iris nozzle channel in this design features a minimum dimension of 10 mm with a blade spacing of 1 mm. Under normal operating conditions, this configuration permits the smooth passage of most aggregates. Furthermore, the blade motion has been optimized to generate controlled turbulence during opening and closing cycles, thereby mitigating aggregate accumulation and blockage.

**Figure 3:**
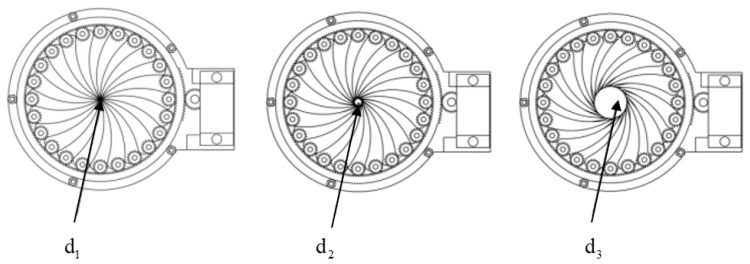
Schematic diagram of the caliber change process of the circular nozzle.

The circular nozzle is adjustable from 0 mm to 40 mm in caliber. The nozzle size adjustment range is illustrated in Figure 3. To prevent corrosion caused by direct contact between the pinion gear and concrete, both the pinion gear and rotating blades are manufactured from 304 stainless steel, which offers excellent durability. Chemically, the chromium content forms an oxide film, ensuring stability in complex chemical environments. Mechanically, it exhibits high strength and toughness, withstanding impact and friction, ensuring that blades resist deformation and damage during prolonged opening and closing cycles. Design-wise, optimized blade structures minimize stress concentration, while proven manufacturing techniques guarantee stable and reliable component connections. Precision engineering reduces clearance gaps, and flange covers incorporate collars to prevent concrete overflow and blockages. Following each print cycle, components are disassembled for cleaning and lubricated with anti-corrosion oil to ensure sustained, stable equipment operation.

## 3. Fluid–Solid Coupling Analysis

The rotary vane is a part of the reducer nozzle that needs to be emphasized, and its design and manufacture directly affect the forming quality of the printed components. The deformation and stress distribution of the rotary blade is the key to the structural design of the rotary blade, so it is necessary to carry out fluid–solid coupling analysis to determine the deformation of the rotary blade under different calibers. Secondly, the structural stress analysis of the rotary blade is also needed to reduce the fatigue damage of the rotary blade and ensure the structural strength and safety of the designed rotary blade.

**Figure 4:**
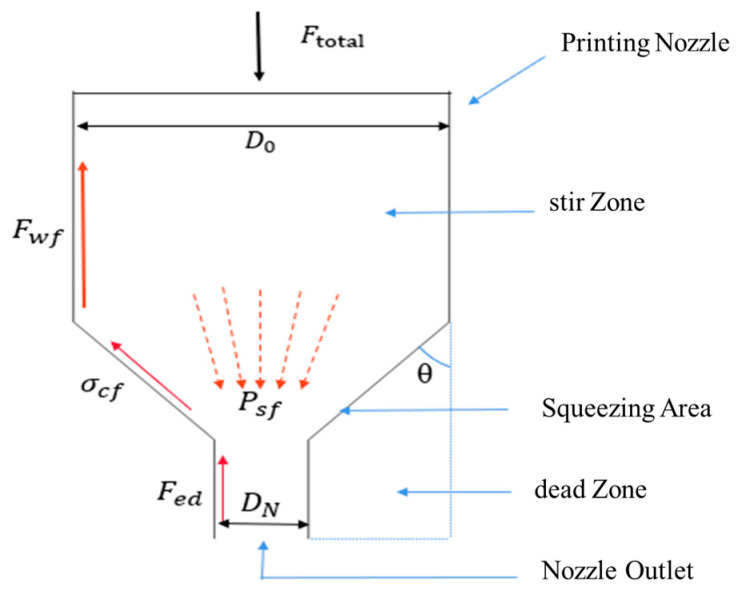
Schematic diagram of the resistance acting on the fluid flowing in the nozzle.

In practical printing, the extrusion resistance encountered by the cement paste in the printing nozzle must be reduced in order to obtain high-quality prints [17]. These resistances consist of four main aspects (as shown in Figure 4), including barrel wall friction $F_{wf}$. The contact between the cement paste and the inner wall of the mixing drum generates friction $F_{ed}$. To minimize this resistance, mixing drums should be designed to be shorter or made of materials with smooth surfaces and low coefficients of friction. If an additional model is provided at the nozzle outlet, the inner surface of the model will generate additional friction on the slurry. The model should be made of low-friction material, and its design should be optimized to reduce the contact area. In terms of dead zone resistance, the dead zone is formed by the flow in the mixing drum, including the molding pressure $P_{sf}$ and cone friction stress $\sigma_{cf}$. They involve the cone angle of the dead zone θ. This requires that the mixing drum design needs to be optimized, e.g., by adjusting the layout of the mixing blades to promote uniform flow of slurry and reduce the dead zone. In terms of counterpressure resistance, in adjustable caliber nozzles, if the outlet caliber is smaller than the maximum caliber, counterpressure resistance will be generated. When used, it should be ensured that the nozzle outlet caliber is the same as the maximum caliber to eliminate this resistance [18]. In summary, optimizing the mixing barrel and nozzle design, using low-friction materials, and setting the nozzle caliber reasonably is the key to reducing extrusion resistance and improving print quality.

### 3.1. Rheology

The rheological properties of concrete exhibit significant variation under different conditions such as ambient temperature, admixtures, and water–cement ratio. Four commonly studied rheological models are the Bingham fluid, Herschel–Bulkley fluid, power-law fluid, and Newtonian fluid [19]. Testing was conducted using a mortar rheometer, specifically the LBY-III model manufactured by Shanghai Rongjida Instrument Technology Co., Ltd., Shanghai, China, to evaluate the rheological properties of Portland cement concrete. The rheometer was preheated for 30 min prior to testing. The measuring system comprised a coaxial cylindrical structure with an outer cylinder caliber of 50 mm, an inner cylinder caliber of 46 mm, and a measuring gap of 2 mm. The experimental temperature was maintained at (20 ± 1) °C. Freshly mixed concrete samples were slowly poured into the test vessel to minimize air entrainment. The test employed a continuous increasing shear rate mode, commencing at 0.1 s^−1^ and increasing by 0.1 s^−1^ increments up to 100 s^−1^. Each rate point was maintained for 60 s, with data recorded upon stress stabilization. Compared with other rheological models, the power-law rheology model offers the advantages of fewer parameters and simpler calculations, enhancing the efficiency of fluid–structure interaction modelling while maintaining computational accuracy. Furthermore, this model effectively accommodates the dynamic opening and closing process of the blades in this nozzle design, accurately describing the flow characteristics of concrete under varying blade openings. This provides a reliable theoretical basis for optimizing the nozzle design. By fitting the experimental data using the least squares method, the fitting parameters and fitting errors for each model were obtained. The specific results are shown in Table 2.

(1) $$\tau =K{\dot{\gamma }}^{n}$$

(2) $$\tau =\tau_{0}+\mu \dot{\gamma }$$

(3) $$\tau =\tau_{0}+K{\dot{\gamma }}^{n}$$

The fitting results indicate that the R^2^ value of the power-law rheological model is 0.98, demonstrating that this model adequately describes the rheological behavior of Portland cement concrete within the shear rate range of this experiment. The rheological equation for the cement paste was derived by fitting the rheological curves.

(4) $$\tau =1.68{\dot{\gamma }}^{0.53}$$

τ denotes shear stress; $\dot{\gamma }$ denotes the shear rate.

### 3.2. Mathematical Modeling

It is assumed that the slurry filling the channel can be regarded as a generalized Newtonian fluid, which is an incompressible isothermal steady state flow, according to rheological theory. Because the viscous force of the slurry is much larger than the inertial force, the inertial force can be ignored. The power law rheological model equations of the slurry flow obeys the continuity and momentum equations as follows:

(5) $$\rho \frac{Du}{Dt}=-𝛻P+𝛻\tau +\rho g$$

(6) 𝛻τ−𝛻PI=0

where u is the velocity vector, q is the fluid density, g is the acceleration of gravity, and I is the unit tensor.

### 3.3. Mesh Division of Mixing Drum

**Figure 5:**
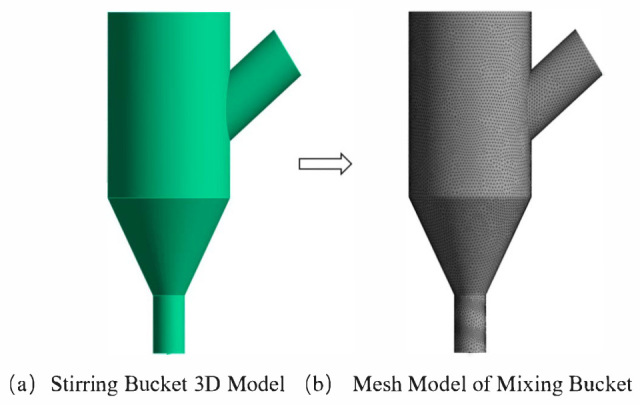
Fluid domain model of stirring region.

When dividing the fluid model in the mixing region, a tetrahedral mesh is used for division, and a hexahedral mesh is used to set the face region near the screw part. The specific division is shown in Figure 5. A total of 872,774 grid cells and 158,388 nodes are divided.

**Figure 6:**
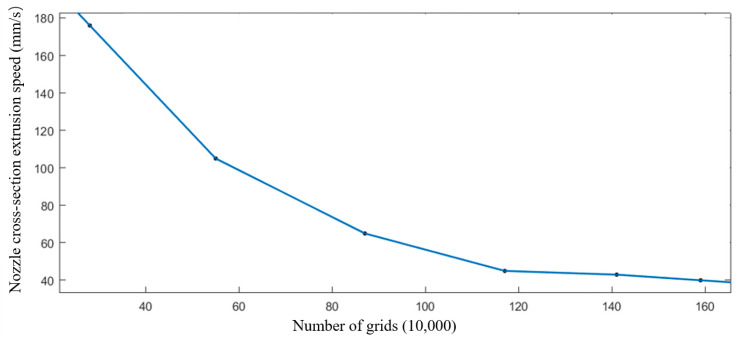
Grid independence verification.

This study employed six distinct mesh configurations to partition the flow channel and rotating domain at the rated rotational speed. The calculations shown in Figure 6 indicate that when the mesh count falls below 870,000, significant variations in outlet velocity occur, leading to inaccurate outcomes. Conversely, when the mesh count exceeds 870,000, outlet velocity changes become negligible. Considering the pronounced fluctuations observed at higher mesh densities, we attribute this phenomenon to numerical instability. Further increases in mesh density may amplify numerical errors, inducing abnormal fluctuations in results. In certain instances, excessive refinement may introduce additional numerical noise, thereby diminishing simulation accuracy. Therefore, after weighing computational cost, result stability, and accuracy, 870,000 meshes is deemed a reasonable choice under current research conditions. As shown in Table 3, results at this mesh size exhibit relative accuracy, with numerical outcomes becoming independent of mesh scale.

### 3.4. Boundary Conditions

**Figure 7:**
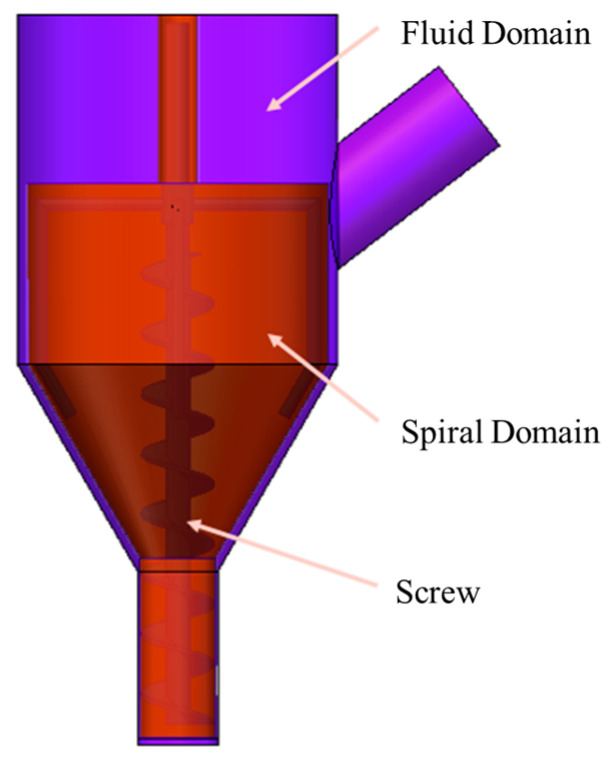
Basin division of mixing tank.

The cement slurry used in the experiment is obtained from a variety of mixed materials, so it is selected as an incompressible multiphase flow model. In the actual work process, one needs to consider the role of gravity. In Figure 7, the mixing barrel was divided into the fluid domain, rotating domain, and fluid domain. The rotating domain of the intersection between the interface was set as the interface boundary. It is preferable to set the rotational speed at 60 rad/min and the boundary conditions of the inlet velocity at 20 mm/s. Standard atmospheric pressure should be used. Turbulence intensity should be set to 5%. The hydraulic caliber is 80 mm, the outlet is set to pressure outlet, and the outlet pressure is set to 0.1 MPa. In the process of extrudate flow, the analysis is carried out in accordance with the ideal situation, ignoring the friction between the inner wall of the nozzle and the fluid and assuming that the wall surface has no slip [20]. After setting the boundary conditions, the SIMPLE solution method is selected to initialize the flow field to simulate the model for simulation calculation.

### 3.5. Mouthpiece Fluid Domain Settings

**Figure 8:**
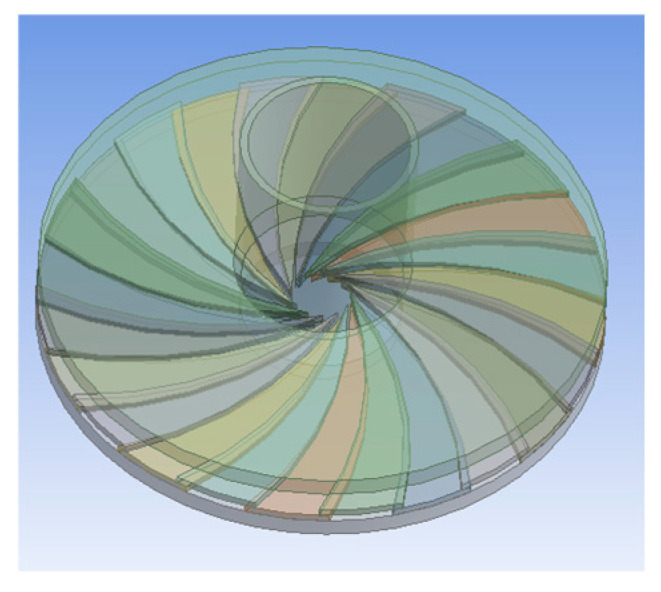
Radiused nozzle model sketch.

This study focuses on the case of rotating blade deformation of a variable-caliber nozzle, and the simplified model is shown in Figure 8.

For the complex flow field in the nozzle, a tetrahedral mesh is used to optimize the blade area, and a hexahedral mesh is used to deal with the rest of the area, with a mesh size of 1.4 mm, a total number of meshes of 255,240, an average mesh quality of 0.859, and a mesh criterion of 0.21. The increase in the mesh density does not have a significant effect on the results, and it is verified that the simulation is close to the actual situation. The unified initial value algorithm is proposed, and the sub relaxation coefficient, time step, and iteration number are set finely to balance the convergence and time cost. The inlet velocity is set to be the flow velocity of the flow field, the output boundary is the outflow, and 1000 iterations are performed to verify the results.

### 3.6. Nozzle Solid Field Settings

**Figure 9:**
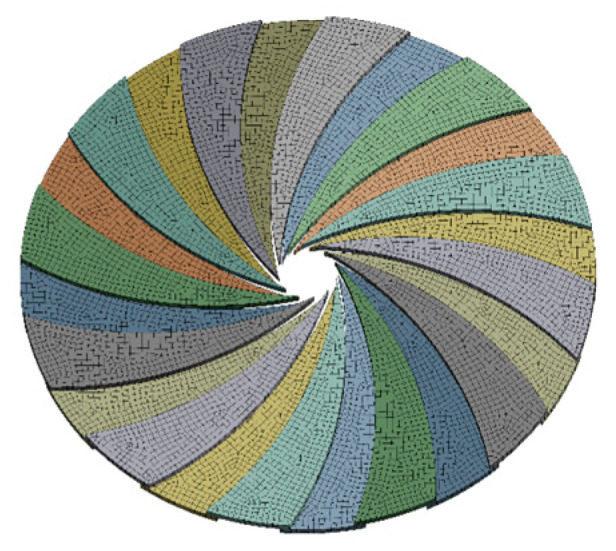
Solid domain meshing of rotating blades.

After simplifying the nozzle, the solid domain part is meshed, and a tetrahedral mesh is selected with a mesh set size of 1.4 mm, as shown in Figure 9.

Sub-material property settings, load, and constraint surface settings are shown in Table 3. The rotating blade material is 304 stainless steel. The specific parameters are shown in Table 4. The outlet caliber of the proposed variable range is = 10~40 mm.

The bottom of the rotor blade ignores the reaction of the deformed blade, and the pressure load obtained from the flow field calculation is iterated internally [21]. When the calculation reaches the critical value, the pressure load is applied to the blade to obtain the blade deformation result at the corresponding moment. Then, we continue to carry out the next time-step solving, and the model diagram is shown in Figure 9 after the completion of the structural parameter settings of the blade. By processing the stress field distribution on the blade surface obtained from the flow field analysis, it is used as the force boundary condition in the transient analysis of the structure and applied in the finite element calculation to realize the solution of the transient response of the blade.

### 3.7. Analysis of Simulation Results

#### 3.7.1. Internal Flow Field Analysis

The numerical simulation of the flow field change caused a change in test caliber under the uniform screw conveying speed. A total of eight different caliber size models (with a specific caliber size of 5 mm, 10 mm, 15 mm, 20 mm, 25 mm, 30 mm, 35 mm, and 40 mm+) were used in the simulation to obtain the flow rate and stress distribution of the nozzle outlet under different calibers and to analyze the trend of the change in the flow field.

**Figure 10:**
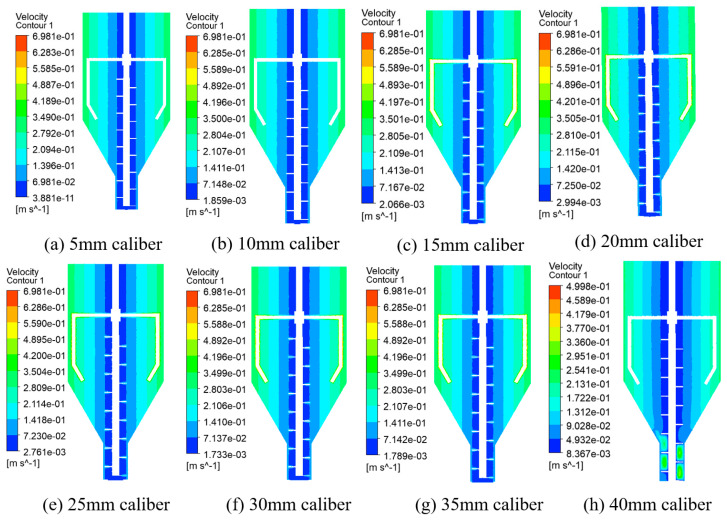
Velocity contours in the fluid region in the mixing module for different calibers.

Figure 10, above, shows different calibers under the mixing module in the fluid area velocity contour map. From the figure, it can be seen that in the mixing barrel, the fluid flow velocity is mostly in 10 mm/s~500 mm/s because the nozzle is symmetrically arranged to the mixing barrel, which is a regular rotating body. Thus, the flow velocity from the wall to the center of the mixing barrel is also uniformly reduced, conveying the smallest screw flow velocity amplitude. The flow velocity in the barrel becomes higher at the blade because the impeller and screw are undergoing coaxial movement. The faster the flow velocity, the more fully the concrete slurry is mixed. The screw mainly plays the role of conveying, and the flow velocity is relatively low. From this point of view, due to the fact that the nozzle caliber continues to expand, the flow rate at the outlet from Figure 10a–h is sequentially lower. This is because the overall flow rate of the mixing drum does not change. When the caliber is expanded, i.e., the unit cross-sectional area is expanded (which will reduce the unit flow rate), the flow rate at the nozzle is sequentially lower.

**Figure 11:**
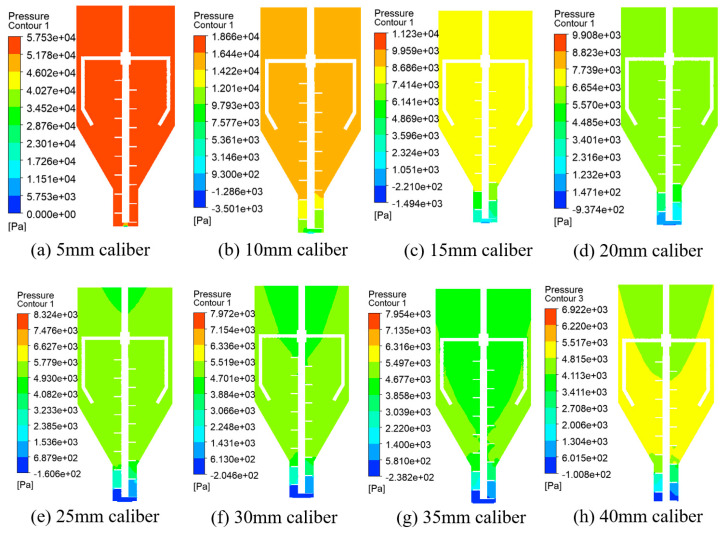
Stress contours in the fluid region in the mixing module at the same caliber.

The contour plots of the stress in the fluid region in the stirring module under different calibers are shown in Figure 11. From the overall viewpoint, the stress in the stirring barrel is decreasing in Figure 11a–h because the flow rate at the outlet increases as the caliber is continuously enlarged, resulting in a decrease in the internal stress in the stirring barrel. Partially, from Figure 11e–h, it can be seen that the macroscopic distribution of stress in the mixing barrels with different calibers is similar, and the stress gradient increases and then decreases along the screw axis when the extrusion outlet is certain; i.e., the overall stress above the mixing barrels is large and then gradually decreases downwards. Near the screw, there is a “valley-like” stress gradient, which is caused by the increased resistance of the extrusion front end due to the outlet size being smaller than the caliber of the mixing barrel. However, Figure 11a–d show the opposite, with no significant change in the stress gradient along the axis of the barrel, indicating that a small outlet caliber can cause high pressure in the barrel, which is not conducive to the extrusion of the slurry. Overall, in comparison in can be seen that because the rotational speed is the same, the bottom outlet caliber is different. In the smallest caliber, the maximum stress is generated in the mixing barrel. As the caliber becomes larger, the stress gradient in the mixing drum gradually decreases.

#### 3.7.2. Structural Mechanical Analysis

The flow field variations, including stress and velocity variations, generated by the arrived mixing drums of different calibers are imported into the solid domain of the rotating blades after the setup is completed, and the diagrams of the blades concerning the shape variations and stress variations are obtained and analyzed.

**Figure 12:**
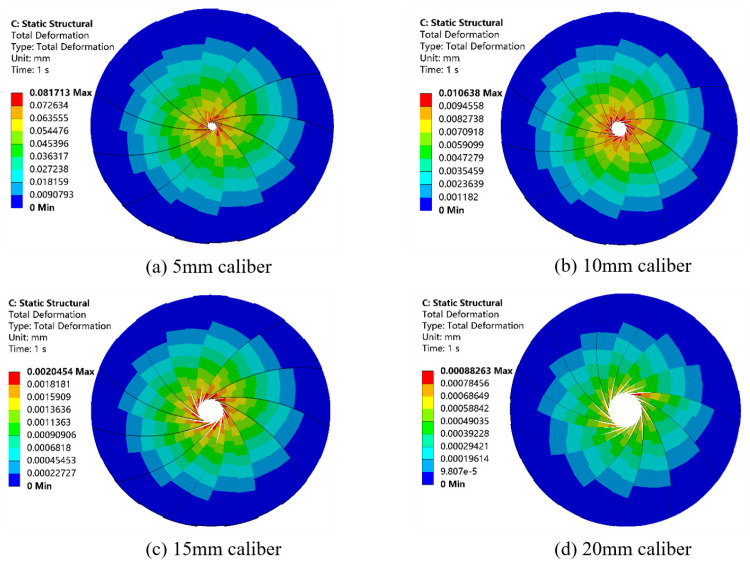

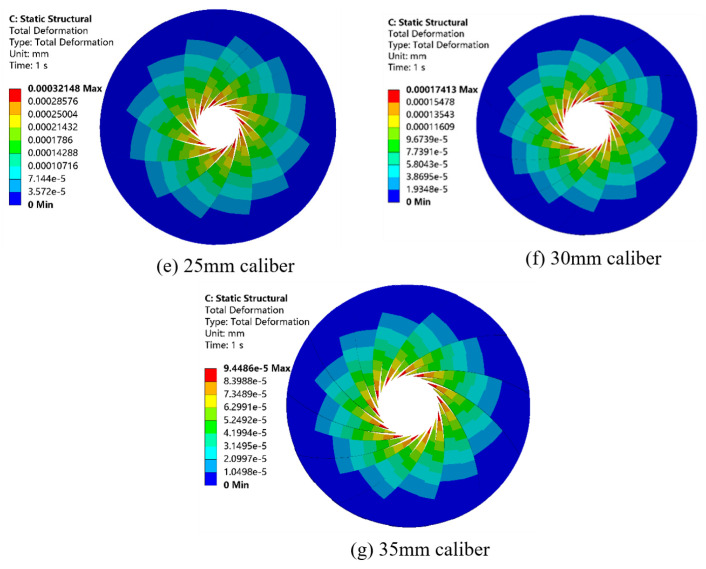
Deformation produced by rotating blades of different caliber at the same inlet velocity.

Figure 12 shows the contour plots of the total deformation distribution of the rotating blades in the outlet caliber of 5, 10, 15, 20, 25, 30, and 35 mm at a certain inlet flow rate. From the individual deformation diagram, it can be seen that the degree of deformation of each blade from the fixed end to the tip of the total deformation of the gradient distribution is mainly concentrated in the middle of the blade to the tip of the part because the root part of the leaf is applied to the fixed constraints, and the cross-sectional area compared to the tip of the leaf in the face of the same external load impact change is relatively small.

From observing Figure 12, it can be seen that overall, the value of deformation increases positively with the decrease in caliber. The maximum deformation is in the caliber of 5 mm; the specific deformation distance is 0.08 mm; the caliber is 35 mm when the total deformation of the blade is the smallest. In different calibers, the maximum and minimum deformation position of the blade and the overall trend of the change did not change too much due to the fact that the internal stress is unchanged, and the caliber is expanded, so that the blade subjected to deformation. Because the internal stress remains unchanged while the expansion of the caliber makes the deformation stress on the blade become smaller, the deformation amount also becomes smaller.

Therefore, the size of the caliber affects the value of the total deformation of the blade, and the difference in the position of the total deformation of the blade also depends on its own structure.

**Figure 13:**
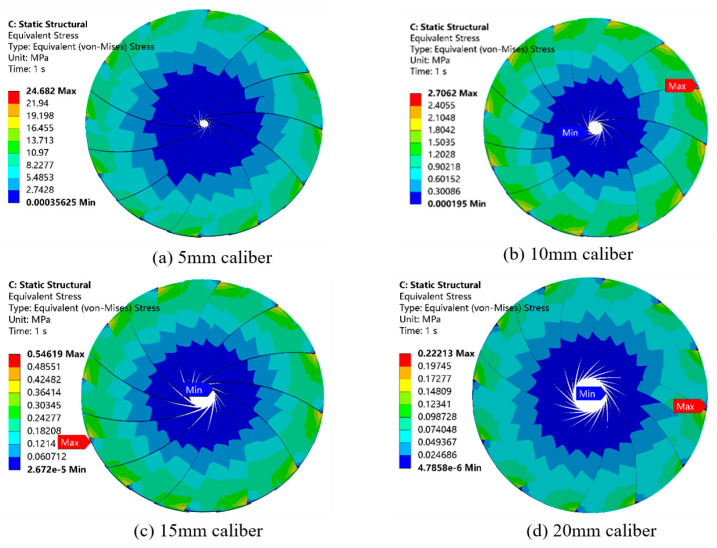

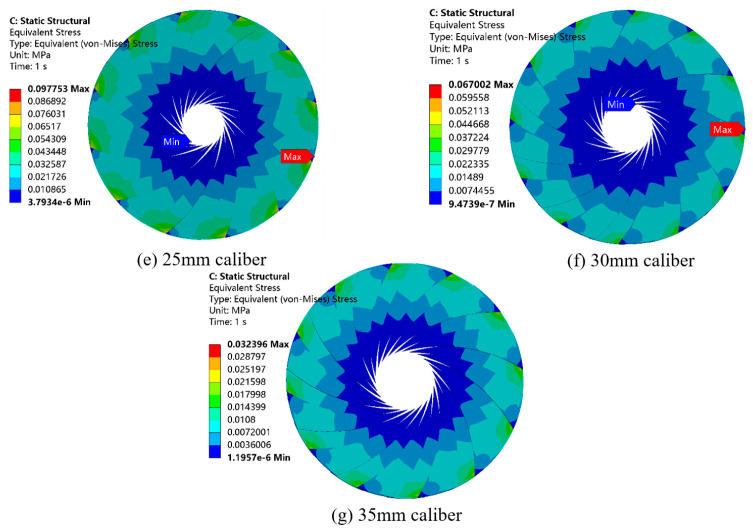
Equivalent forces generated by rotating blades of different calibers at the same inlet velocity.

Figure 13 shows the contour plot of the distribution of the equivalent force of the rotating blade in the caliber of 5, 10, 15, 20, 25, 30, and 35 mm, respectively, when the inlet speed is the same. From observing Figure 13, overall, it can be seen that with the increase in caliber, the equivalent force on the surface of the blade decreases. When the caliber is 35 mm, the maximum equivalent force of the blade is 0.03 MPa. When the caliber is 5 mm, the maximum equivalent force of the blade is 24.61 MPa, and the equivalent force is about 820 times that of the caliber (35 mm).

From the partial diagram in Figure 13, it can be seen that there is an obvious pressure gradient from the middle of the blade to the side. The stress distribution of the blade surface is uniform under different calibers, and the stress is larger near the fixed position, while the stress at the tip position of the blade is smaller because the position of the stress concentration is the position of the largest force when the blade is rotating, which corresponds to the deformation of the blade. The back end of the blade is fixed, which leads to deformation at the tip of the blade when the cement slurry is extruded. This will create the force generated at the rear end of the blade [22]. Therefore, the surface of the rotating blade is affected in a non-negligible way.

**Figure 14:**
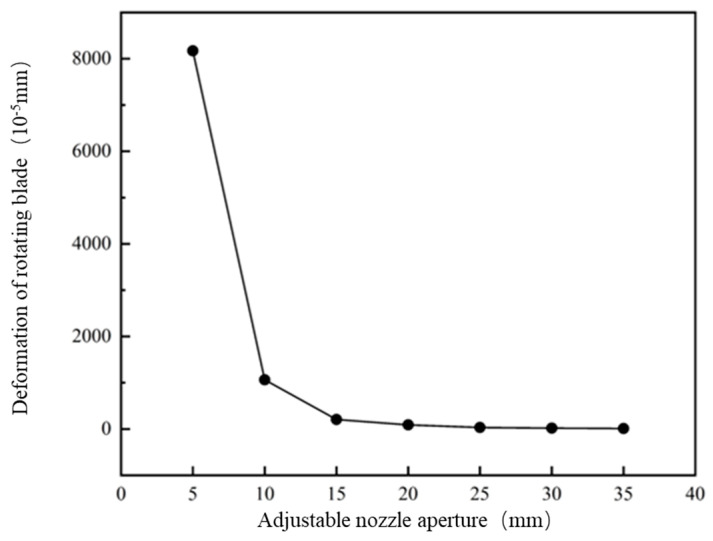
Deformation curves of rotating blades with the same inlet velocity and different calibers.

Following analysis of Figure 14, above, which shows a deformation line graph of different caliber rotary blades, it can be seen that for the caliber of 5 mm~10 mm, the tip of the rotary blade deformation changes significantly, by about 0.08 mm, because the smaller the caliber, the larger the mixing barrel internal stress is. Thus, for the blade to withstand the ability to be large, the deformation change must be relatively large: in the 15 mm~35 mm range. When the rotary blade deformation change is very small due to a caliber increase, the mixing is in the 15 mm~35 mm range. The variation in the rotary blade deformation is very small because with the increase in caliber, the internal stress of the mixing barrel decreases, and the stress on the blade also decreases. In this paper, we used 10 mm~40 mm caliber deformation. With a caliber of 10 mm, the maximum deformation of the rotary blade variable is 0.01 mm. Its deformation changes will not affect the print quality, so the blade design meets the requirements.

**Figure 15:**
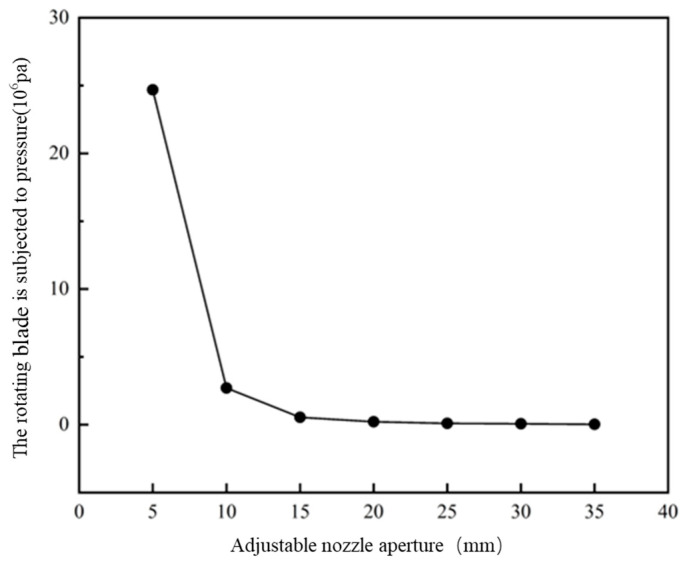
Equivalent pressure curves of rotating blades with the same inlet velocity and different calibers.

Following analysis of Figure 15, above, which shows an equivalent force line graph for different caliber rotating blades, it can be seen that for the caliber of 5 mm~10 mm, the equivalent force of the blade changes significantly: by 10 mm~15 mm when the deformation changes 13 times. As the overall flow rate is unchanged, the caliber continues to reduce, the unit area flow rate increases, and the stronger the coupling between the fluid and the solid is. The blade is able to withstand the equivalent increase, making the fluid on the surface of the blade play a greater role.

## 4. Printing Test Effects

**Figure 16:**
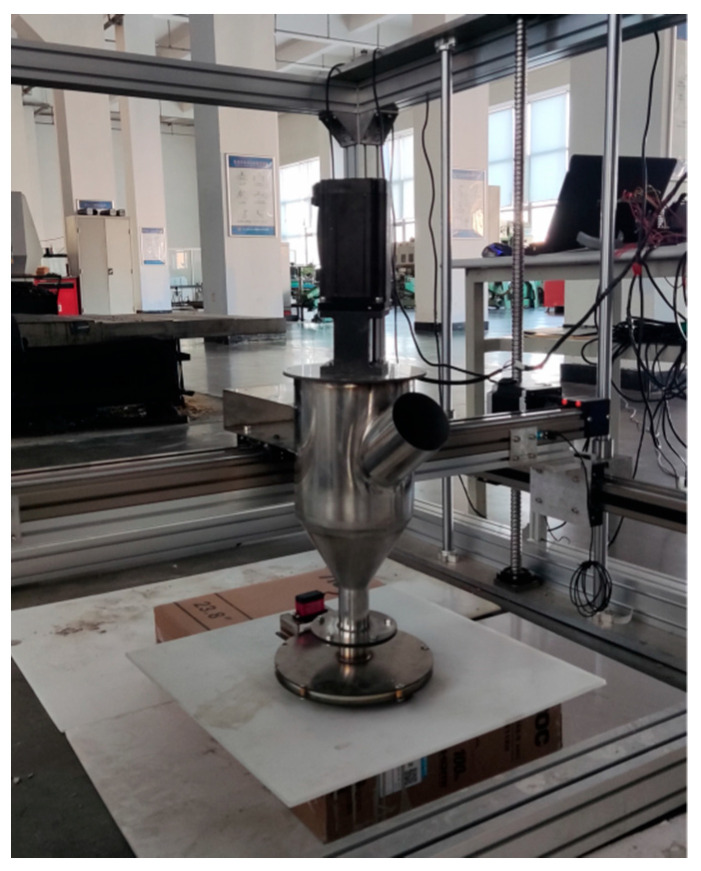
Physical diagram of concrete 3D printing experiment platform.

The adjustable printing nozzle is placed on the printing device platform that already exists in the laboratory. The concrete 3D printer XYZ axis motion system, control system, and extrusion system are included. The specific physical diagram is shown in Figure 16. The drive XYZ axis motion system is driven by an 86-stepper motor. The experimental material is rheological cement paste.

Printing was carried out at a room temperature of 20 °C. A circular nozzle with a print caliber of 10 mm was used for fixed-caliber printing; the height of the print layer was 6 mm, the number of print layers was one layer, the print speed was 20 mm/s, and the screw extrusion speed was 40 r/min.

**Figure 17:**
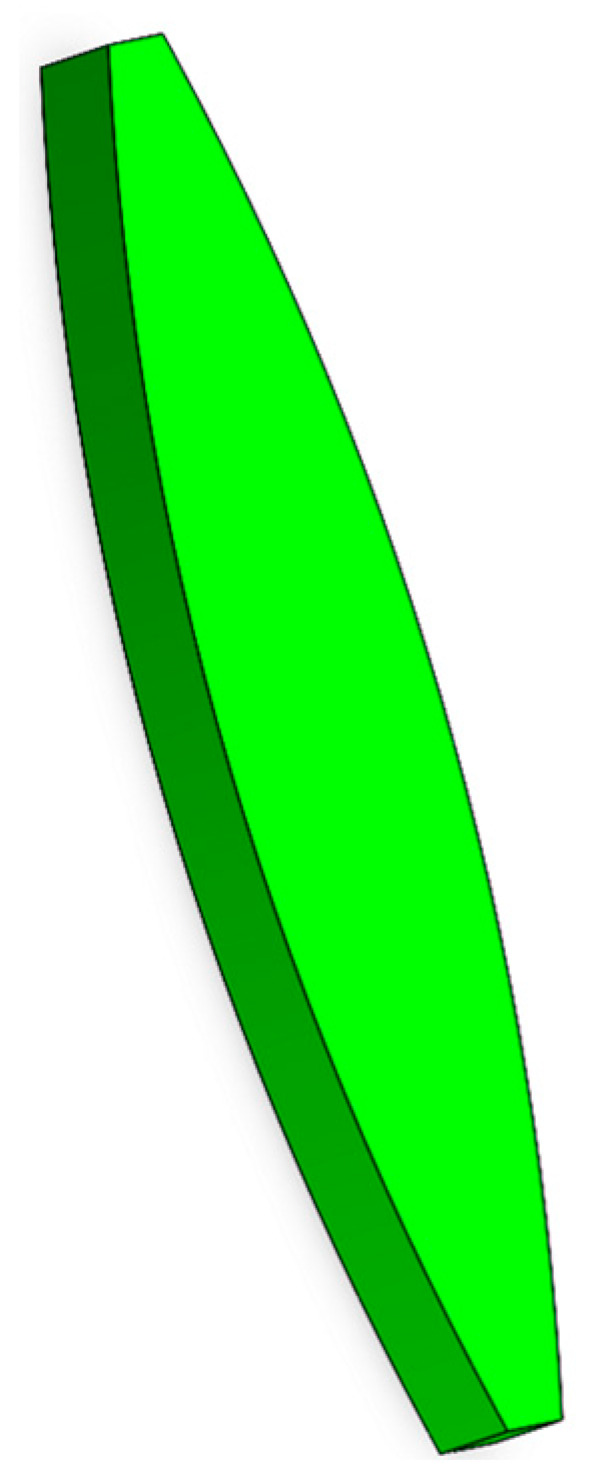
3D test printing of component models.

Firstly, it is necessary to weigh 500 g of silicate cement, 500 g of ordinary sand, 200 g of water, 2 g of cellulose, and 7 g of coagulant promoter and mix them thoroughly with water; the printing model is shown in Figure 17, and the fixed-caliber nozzle is printed by the method of parallel straight line printing and contour offset printing.

**Figure 18:**
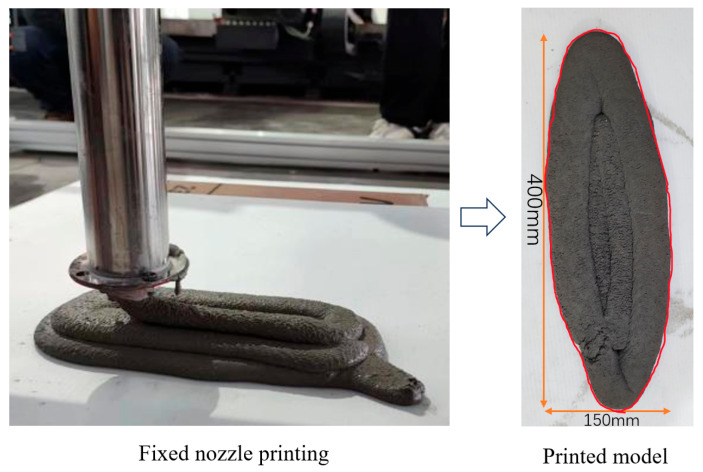
Print component sample.

The print result is shown in Figure 18.

**Figure 19:**
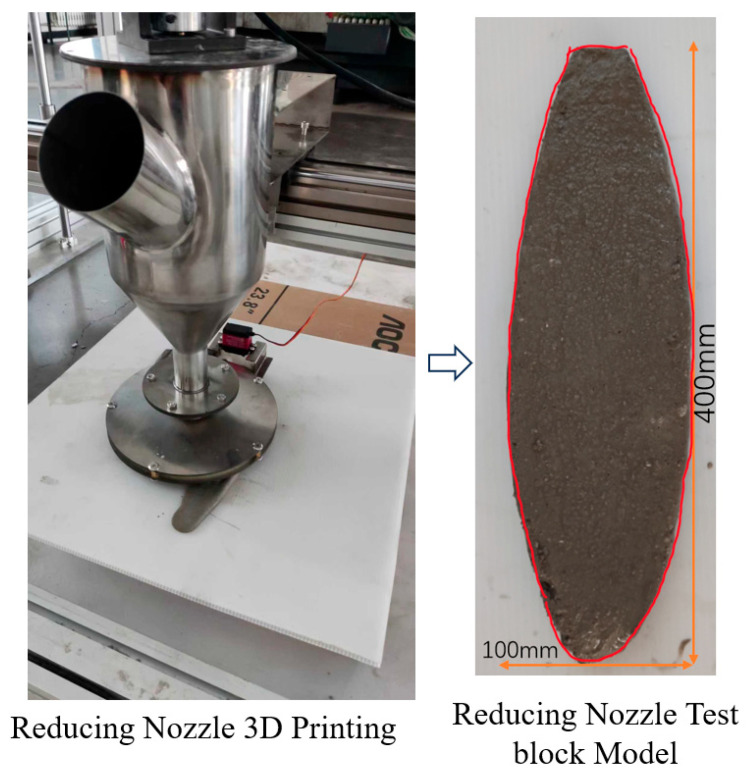
Reducing nozzle printed component samples.

From the adjustable nozzle printing component samples in Figure 18, it is clear that the adjustable nozzle printing components significantly improve the poor molding quality of parallel line printing and contour printing “overfill” and “underfill” problems. As demonstrated by the printed component samples from the adjustable nozzle in Figure 19, it is evident that the adjustable nozzle significantly improves the poor forming quality of parallel straight-line printing, as well as the “overfilling” and “underfilling” issues observed in contour printing. Visual comparisons were conducted on printed samples during the experiment. From an intuitive visual perspective, specimens printed using this variable-caliber nozzle exhibited a flatter and smoother surface appearance compared to those printed with existing concrete 3D printing nozzles. Superior performance was observed in surface roughness, dimensional accuracy testing, layer thickness consistency analysis, and mechanical properties of the printed specimens. Based on the variable-caliber nozzle’s centering mechanism, precision adjustment via gear meshing, and detachable variable-speed design principles, it is evident that this nozzle configuration may enhance surface roughness performance in practical applications.

Analyzed through the comparison results, the components printed with adjustable nozzles obviously eliminate the “overfill” and “underfill” problems that occur in parallel linear printing and contour printing. This verifies that the adjustable nozzle and variable-caliber path designed in this paper have some significance in concrete 3D printing.

## 5. Conclusions

This paper describes a variable-caliber nozzle using multiple gears with internal meshing and rotating blade structure, aiming to improve the surface quality of concrete 3D specimens. The printing stroke is shortened, and the printing accuracy is improved. The completion of the content specifically includes the following:

1. Multi-tooth internal meshing print nozzle for printing components for multiple cycles of repeated printing. This innovation is also different from the existing adjustable nozzle. A multi-tooth internal meshing print nozzle can be used, depending on the size of the rotating blade torque transfer, to realize the nozzle’s adjustment in order to improve the specimen’s surface quality and improve the printing of the “overfill” and “underfill” problems.
2. Through the hydrodynamic simulation analysis of the rotary blade form variable and stress diagram, it is concluded that in the caliber of 10 mm, the form variable is 0.01 mm, which is less than the engineering roughness of 0.05 mm. Finally, the maximum value of the stress obtained is 2.7 MPa, which is less than the yield strength of stainless steel. And the printing accuracy and the strength of the rotary blade material are in accordance with the standard, so the structural strength and safety of the designed rotary blade is qualified.
3. Through a comparative analysis of the results of using a fixed-caliber nozzle and variable-caliber nozzle on the same model for test-piece printing, it can be observed that using an adjustable nozzle to print components obviously eliminates the parallel straight-line printing of the print path, including the problem whereby it is too long and the problem whereby contour printing of the “overfill” and “underfill” occurs. This verifies that the variable-caliber nozzle designed in this paper has some significance in concrete 3D printing.

Concrete 3D printing is an important development direction of the new building additive manufacturing technology, for the realization of the functionality of complex profile components is of great significance, and I hope that in the future, some of the problems can be further improved and can be carried out to expand the following aspects:

1. In this paper, for the development of concrete 3D printing equipment and technology, we designed a variable-caliber nozzle and verified the feasibility of the equipment, but the maximum caliber was 40 mm, which restricted the printing function. We need to further expand its printing of the cross-sectional area and study the mechanism that is more suitable for industrial applications.
2. The gear engagement part of the current reducer nozzle is built by setting up the microcontroller program first and additionally controlling the motor at the start of printing to achieve the print caliber size adjustment, and it is necessary to develop a separate program for controlling the servo in parallel with the software at a later stage.

## Figures and Tables

**Table 1:** Main parameters of adjustable printhead.

Parameters	Value
Nozzle caliber (mm)	220
Adjustable-print nozzle caliber (mm)	10~40
Rotary blade thickness (mm)	2
Number of rotating blades (pieces)	24

**Table 2:** Rheological Equation.

Model	Equation	R^2^
Power law	K=1.68, n=0.53	0.98
Bingham	$\tau_{0}=5.2$ Pa, μ=8.6 Pa·s	0.90
Herschel–Bulkley	$\tau_{0}=4.8$ Pa, K=11.8, n=0.88	0.93

**Table 3:** The grid number of model and outlet velocity.

Group	1	2	3	4	5
Number of grids	165,765	369,701	560,571	701,598	869,167
Export speed (mm/s)	162.3	91.4	73.48	45.21	42.3

**Table 4:** Material parameters.

Material Property	Numerical Value
Elastic modulus E (GPa)	206
Poisson’s ratio	0.3
Yield stress (MP_a_)	170
Thickness (mm)	2

## Data Availability

The original contributions presented in this study are included in the article. Further inquiries can be directed to the corresponding author.
